# Posttranscriptional Gene Regulation by Spatial Rearrangement of the 3′ Untranslated Region

**DOI:** 10.1371/journal.pbio.0060092

**Published:** 2008-04-29

**Authors:** Andrea B Eberle, Lukas Stalder, Hansruedi Mathys, Rodolfo Zamudio Orozco, Oliver Mühlemann

**Affiliations:** Institute of Cell Biology, University of Berne, Berne, Switzerland; University of Wisconsin, United States of America

## Abstract

Translation termination at premature termination codons (PTCs) triggers degradation of the aberrant mRNA, but the mechanism by which a termination event is defined as premature is still unclear. Here we show that the physical distance between the termination codon and the poly(A)-binding protein PABPC1 is a crucial determinant for PTC recognition in human cells. “Normal” termination codons can trigger nonsense-mediated mRNA decay (NMD) when this distance is extended; and vice versa, NMD can be suppressed by folding the poly(A) tail into proximity of a PTC or by tethering of PABPC1 nearby a PTC, indicating an evolutionarily conserved function of PABPC1 in promoting correct translation termination and antagonizing activation of NMD. Most importantly, our results demonstrate that spatial rearrangements of the 3′ untranslated region can modulate the NMD pathway and thereby provide a novel mechanism for posttranscriptional gene regulation.

## Author Summary

Correct expression of the genetic information is essential for life, and several quality control systems have evolved to ensure accurate protein synthesis. One of these processes, termed nonsense-mediated mRNA decay (NMD), detects inappropriate termination of mRNA translation at premature termination codons (PTCs) and triggers degradation of the aberrant mRNA. Although the occurrence of NMD is well documented in yeast, worms, flies, mammals, and plants, the mechanism by which a termination event is defined as premature is still unclear, and different models have been proposed for different species. For mammals, the current prevailing view is that a termination codon is identified as premature and elicits NMD when it is located upstream of the 3′-most exon junction complex. However, well-documented examples of NMD triggered by PTCs in the last exon challenge this “mammalian NMD model.” Here we show that the physical distance between the termination codon and the poly(A)-binding protein PABPC1 is a crucial determinant for PTC recognition in human cells, indicating an evolutionarily conserved function of PABPC1 in promoting correct translation termination and antagonizing activation of NMD. Most importantly, our results demonstrate that spatial rearrangements of the 3′ untranslated region can modulate the NMD pathway and thereby provide a novel, translation-dependent mechanism for posttranscriptional gene regulation.

## Abstract 3

The physical distance to the poly(A) tail is a crucial determinant to define a termination codon as premature in human cells. This indicates evolutionary conservation of the basic mechanism of nonsense-mediated mRNA decay and provides a novel mechanism for translation-dependent posttranscriptional gene regulation.

## Introduction

Nonsense-mediated mRNA decay (NMD) represents a translation-dependent posttranscriptional mRNA quality control process that selectively degrades mRNAs containing premature termination codons (PTCs), thereby preventing the synthesis of truncated, potentially deleterious proteins [1,2]. Because one-third of all known disease-causing mutations are predicted to generate a PTC, NMD serves as an important modulator of genetic disease phenotypes in humans [3,4]. Hence, understanding the molecular mechanism of NMD will facilitate the future development of gene-specific therapies for many genetic diseases. Interestingly, NMD affects 3%–10% of the transcriptome of Saccharomyces cerevisiae, Drosophila melanogaster, and mammals, indicating that NMD, in addition to its quality control function, is also involved in regulating the expression of many physiological transcripts (reviewed in [5]).

The three Upf (*Up-f*rameshift) proteins—Upf1, Upf2, and Upf3—work at the heart of the NMD pathway in all organisms studied. The Upf proteins were first discovered in genetic screens in S. cerevisiae and Caenorhabditis elegans, and orthologs have subsequently been identified in other eukaryotes (reviewed in [6]). Upf1 is an ATP-dependent RNA helicase, and a mutation in the ATPase domain abolishes its 5′-to-3′ helicase activity and its function in NMD [7–9]. Human Upf2 contains three conserved middle of eIF4G-like (MIF4G) domains, multiple putative nuclear localization signals in its N-terminus, and a putative nuclear export signal. Upf2 interacts with Upf1 and Upf3, and the three proteins can be isolated as a complex [10–13]. Upf3 is the least conserved component among the Upf proteins [6]. Humans contain two different UPF3 genes, encoding Upf3a and Upf3b (also known as Upf3X since the corresponding gene maps to the X chromosome, respectively) [11,13]. In addition to Upf1–3, metazoans contain additional NMD factors (Smg1, Smg5, Smg6, and Smg7) that are involved in regulating the phosphorylation state and therewith the activity of Upf1 [14].

While the phenomenon of NMD and its impact on gene expression are well documented, the understanding of the underlying molecular mechanisms is still fragmented. A central question is how PTCs are recognized and discriminated from natural termination codons (TCs). The current models for NMD differ remarkably between mammals and other eukaryotes. While all models agree that translation is required for NMD, studies of mammalian genes indicate that NMD in higher eukaryotes also depends on pre-mRNA splicing. The current model for mammalian NMD postulates that PTC recognition requires an interaction between an exon junction complex (EJC) bound to the mRNA downstream of the TC and the terminating ribosome [15,16]. However, several examples of NMD in mammals have been reported that are inconsistent with this EJC-dependent NMD model ([17] and references therein). Recently, we showed that PTCs in the terminal exon of Ig-μ minigenes (miniμ) elicit NMD dependent on the length of their 3′ untranslated region (UTR) [17]. This is reminiscent of the situation in D. melanogaster, C. elegans, S. cerevisiae, and plants, where PTC recognition occurs independently of splicing and EJC factors, but where instead 3′ UTR length and the poly(A)-binding protein (PABP) were found to play an important role [18–20]. The “faux 3′ UTR” model, which is based on studies with yeast, postulates that proper translation termination requires an interaction between PABP and eRF3 bound to the terminating ribosome, and that the absence of this positive signal leads to aberrant termination and NMD as a consequence [21]. We report here that our results obtained with human cell lines are consistent with the yeast “faux 3′ UTR” model, providing strong evidence for an evolutionarily conserved basic mechanism of PTC recognition in which PABPC1 acts as an NMD antagonizing factor and a role for the mammalian EJC as an NMD enhancer. But most importantly, our data show that mRNA half-lives can be regulated by altering the spatial configuration of their 3′ UTRs. This represents a novel, potentially widespread mechanism for posttranscriptional gene regulation by NMD.

## Results

### 3′ UTR Extensions Reduce the mRNA Half-Life by NMD

**Figure 1:**
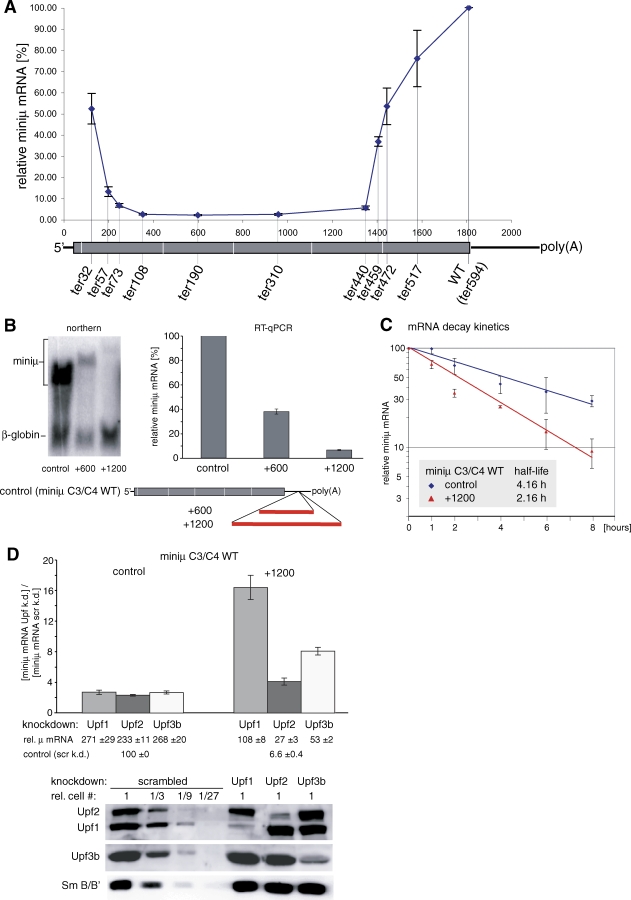
The Distance between the TC and the Poly(A) Tail Is Crucial for NMD (A) Relative miniμ mRNA levels, normalized to the mRNA levels of the cotransfected β-globin WT gene, were determined by RT-qPCR from miniμ constructs with PTCs at the indicated amino acid positions. PTC positions on the mRNA are plotted on the *x*-axis, and average values and SD of four qPCR runs are shown. The miniμ mRNA is schematically shown below the plot with the positions of the PTCs in the different constructs indicated. In all figures, 5′ and 3′ UTRs are depicted as black lines, ORFs as gray boxes, and exon-exon junctions as vertical white lines. (B) Northern blot and RT-qPCR analysis of miniμ mRNA with a 3′ UTR extension by 600 or 1,200 nucleotides. RT-qPCR data were obtained and normalized as in (A). (C) mRNA decay kinetics of the control miniμ C3/C4 WT construct and the +1,200 construct illustrated in (B) were determined in HeLa Tet-Off cells. Relative miniμ mRNAs, normalized to cotransfected β-globin, were analyzed 0, 1, 2, 4, 6, and 8 h after doxycycline addition by RT-qPCR. Average values and SD of two independent experiments with three RT-qPCR runs each are shown. (D) Effect of RNAi-mediated depletion of Upf1, Upf2, or Upf3b on the relative miniμ mRNA levels of the control and the +1,200 construct. The ratio of the normalized miniμ mRNA levels between the indicated Upf knockdown and the control knockdown (scrambled) is represented. Average and SD of three qPCR runs are shown in (B) and (D). The efficacy of the Upf1, Upf2, and Upf3b knockdown was monitored by Western blotting (lower panel). Detection of SmB/B' served as loading control.

Comparison of relative mRNA levels of miniμ constructs with PTCs at different positions indicated that PTCs located toward the 5′ and the 3′ ends of the mRNA induce gradually less efficient NMD (Figure 1A). Because mRNAs were shown to adopt a circular conformation that positions the 5′ end close to the 3′ end by eIF4G bridging the cap-bound factors (eIF4E or CBC) with the poly(A)-binding protein PABPC1 bound to the 3′ end [22–24], and based on our previous results [17], we hypothesized that the distance between the TC and the poly(A) tail might be a crucial determinant to identify a TC as premature. Supporting this hypothesis, extension of this distance by insertion of prokaryotic sequence into the 3′ UTR redefines the normal TC as NMD-triggering PTC (Figure 1B–1D and [17]). Prokaryotic sequence was chosen for these 3′ UTR extensions to minimize the risk of unintentionally inserting binding sites for mammalian RNA-binding proteins that could affect transcript stability. Extending the 3′ UTR of a miniμ construct with the full-length coding region (miniμ C3/C4 WT) from 300 to 900 nucleotides reduced the mRNA level to 40%, and an extension to 1,500 nucleotides led to a further reduction to 7% (Figure 1B). Judged from the Northern blot analysis, insertion of these sequences into the 3′ UTR did not interfere with pre-mRNA splicing or 3′ end formation. We further determined the decay kinetics of the miniμ WT and miniμ WT +1,200 mRNA using the Tet-Off Advanced System in HeLa cells. The 3′ UTR extension of 1,200 nucleotides caused a reduction of the mRNA's half-life from 4.16 h (miniμ WT) to 2.16 h (miniμ WT +1,200; Figure 1C). Additionally, the mRNA reduction of miniμ WT+1,200 can be suppressed to variable extent by RNAi-mediated knockdown of Upf1, Upf2, and Upf3b, indicating that it is caused by bona fide NMD (Figures 1D and S1). Depletion of Upf1 resulted in a 16-fold, depletion of Upf2 in a 4-fold, and depletion of Upf3b in an 8-fold increase of miniμWT+1,200 mRNA, respectively. These differences in the extent of NMD suppression most likely reflect different knockdown efficiencies and/or different minimal concentrations of these proteins required to sustain NMD.

A similar extension of the 3′ UTR of a β-globin reporter gene from 128 to 705 nucleotides (Figure S2), and extension of the miniμ 3′ UTR by a different sequence [17], also caused an Upf1-dependent mRNA reduction, suggesting that the length of the 3′ UTR is an important and general determinant to define a TC as premature and trigger NMD independent of the sequence context. Because in all these examples (i) no exon-exon junction and hence no EJC is located downstream of the PTC, and (ii) several lines of evidence suggest that the upstream EJCs have been removed by the first translating ribosome [25], we refer to this form of NMD as “EJC-independent.”

### Reducing the Physical Distance between the TC and the Poly(A) Tail Suppresses EJC-Independent NMD

**Figure 2:**
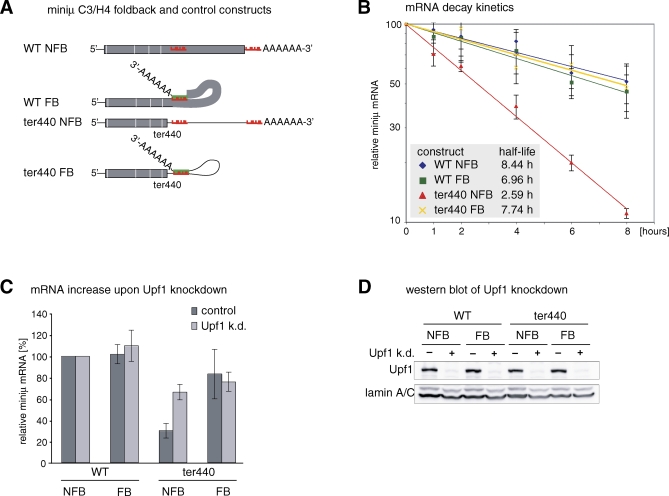
Suppression of EJC-Independent NMD by Poly(A) Tail FB (A) Schematic illustration of the mRNAs expressed by the indicated constructs. The 26-nucleotide sequence located 42 nucleotides downstream of codon 440 is depicted in red, and the insertion upstream of the poly(A) tail of this sequence (red) or of the complementary sequence (green) is indicated. WT = construct with full-length ORF; ter440 = construct with PTC at codon 440. (B) Half-lives of the FB mRNAs were measured as described in Figure 1C. (C) Relative miniμ mRNA levels from the EJC-independent FB constructs shown in (A) normalized to β-globin WT mRNA from a cotransfected plasmid, were measured by RT-qPCR from Upf1-depleted cells (light gray bars) or from control cells expressing a scrambled shRNA (dark gray bars). (D) The efficacy of the Upf1 knockdown was assessed by Western blotting. Detection of lamin A/C served as loading control.

Because many transcripts in higher eukaryotes have long 3′ UTRs [26,27], simply the number of nucleotides between the TC and the poly(A) tail is unlikely to represent the signal for defining a TC as premature. Instead, we hypothesized that it may be rather the physical distance between the TC and the poly(A) tail that bears the kinetic and regulatory potential to distinguish a proper translation termination event from aberrant termination. According to this model, it should be possible to suppress NMD by reducing this distance. We tested this with so-called “foldback” constructs, in which 26 nucleotides complementary to the sequence located about 50 nucleotides downstream of the PTC were inserted into miniμ immediately upstream of the poly(A) signal. Base pairing of this complementary sequence positions the poly(A) tail in the vicinity of the PTC in these transcripts (Figure 2A). Northern blot analysis (Figure S3A) and reverse transcriptase (RT)-PCR (unpublished data) confirmed that introduction of this intramolecular base pairing did not interfere with splicing or 3′ end processing. For this EJC-independent NMD reporter (miniμ C3/H4 ter440 [17]), a mRNA half-life of 2.59 h was observed with the miniμ ter440 “no foldback” (NFB) control construct, whereas the mRNA half-life of the corresponding “foldback” (FB) construct was with 7.74 h very similar to the half-lives of the control constructs WT NFB (8.44 h) and WT FB (6.96 h, Figure 2B). Furthermore, the steady-state mRNA level of the miniμ ter440 NFB control construct was reduced to 30% in an Upf1-dependent manner, whereas the mRNA level of the corresponding FB construct was only marginally reduced compared to the WT and was not affected by RNAi-mediated Upf1 depletion (Figure 2C and 2D). To confirm that the observed NMD suppression of ter440 FB mRNA was specifically dependent on the base pairing between the inserted sequence near the poly(A) tail and the complementary region about 50 nucleotides downstream of ter440, we mutated seven nucleotides in this region to abolish the base pairing potential (ter440 mutFB, Figure S4A). The Upf1-dependent mRNA reduction observed with ter440 mutFB shows that this mRNA is a substrate for NMD (Figure S4B and S4C). This indicates that NMD suppression of ter440 FB requires the actual formation of the predicted intramolecular base pairs, because abolishing of this base pairing potential renders the transcript NMD-sensitive. Collectively, these results demonstrate that folding the poly(A) tail into the vicinity of a PTC in the terminal exon suppresses EJC-independent NMD.

### mRNA Stability of FB Constructs Gradually Decreases with Increasing Distance between TC and Poly(A) Tail

**Figure 3:**
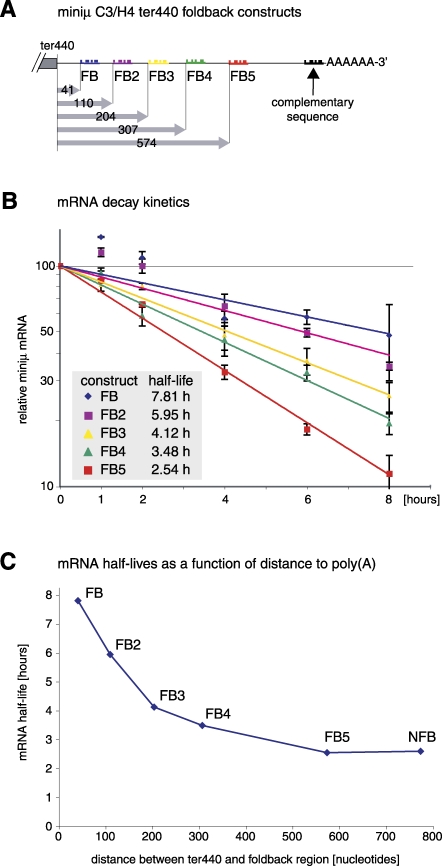
The Distance between the PTC and the Poly(A) Tail Is an Important Criterion for mRNA Stability (A) Schematic illustration of the miniμ C3/H4 ter440 FB mRNAs expressed by the indicated constructs. Complementary sequences to the regions FB, FB2, FB3, FB4, and FB5 were inserted as double-stranded oligonucleotides at the position marked by the arrow. The distance (in number of nucleotides) between the PTC (ter440) and the first base of the respective base pairing region are indicated below. (B) Half-lives of the FB mRNAs were measured as described in Figure 1C. Average values and SD from one experiment with three RT-qPCR runs are shown. (C) Half-lives of the indicated mRNAs (data from Figures 2B and 3B) were plotted against the distance between ter440 and the first base of the base-pairing region.

Next we wanted to determine up to which maximal distance from the TC the poly(A) tail is able to suppress NMD. We generated additional FB constructs analogous to miniμ C3/H4 ter440 FB (Figure 2) by inserting different sequences into the poly(A) signal proximal Spe1 restriction site that are complementary to different regions downstream of ter440 (Figure 3A). The complementary sequences are between 20 and 30 nucleotides long and designed to have a melting temperature of about 60 °C when base pairing to their target sequence (see Materials and Methods). In HeLa cells transiently transfected with these pTRE-tight FB reporter constructs, we stopped transcription by addition of doxycycline and analyzed the decay kinetics of the FB transcripts. These experiments revealed a gradual destabilization of the mRNAs with increasing distance of the poly(A) tail from the PTC (Figure 3B and 3C). Noteworthy, the two independently determined half-lives for miniμ C3/H4 ter440 FB mRNA differ by less than 1% (7.74 h in Figure 2B and 7.81 h in Figure 3B), indicating that these half-life measurements are highly reproducible and precise. Whereas the half-life of FB mRNA is similar to the half-lives of WT NFB and WT FB (Figure 2B), indicating a complete suppression of NMD, the half-life of FB5 (2.54 h, Figure 3B) is similar to the half-life of ter440 NFB (2.59 h, Figure 2B), indicating a complete loss of NMD suppression. The half-lives of FB2, FB3, and FB4 mRNA fall in-between and indicate a partial loss of NMD suppression. From these results, we conclude that the poly(A) tail–mediated NMD-suppressing activity functions in a distance-dependent manner, as manifested by the gradual decrease of mRNA stability upon increasing distance between TC and poly(A) tail (Figure 3C).

### Reducing the Physical Distance between the TC and the Poly(A) Tail Also Suppresses EJC-Enhanced NMD

**Figure 4:**
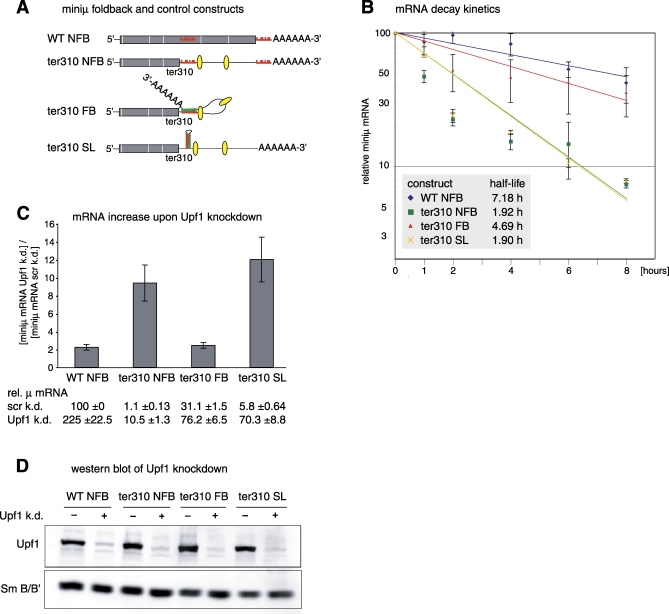
Suppression of EJC-Enhanced NMD by Poly(A) Tail FB (A) Schematic illustration of the mRNAs expressed by the indicated constructs. The 26-nucleotide sequence located 28 nucleotides downstream of codon 310 is depicted in red, and the insertion upstream of the poly(A) tail of this sequence (red) or of the complementary sequence (green) is indicated. Predicted EJCs in the 3′ UTR are shown by yellow ovals. WT = construct with full-length ORF; ter310 = construct with PTC at codon 310; SL = stemloop control construct with complementary sequence inserted in exon C2. (B) Half-lives of the FB mRNAs were measured as described in Figure 1C. (C) Relative miniμ mRNA levels from FB constructs with 3′ UTR introns expressed in cells with (+) or without (−) Upf1 knockdown were measured as in Figure 2C and are shown below the histogram. The histogram depicts the fold increase of miniμ mRNA upon Upf1 knockdown. (D) The Upf1 knockdown efficacy was assessed by Western blotting as in Figure 2D. Detection of Sm B/B' served as loading control.

Our previous results suggested that in mammals, the EJC has adopted a NMD-enhancing function when present downstream of a TC, presumably by increasing the local concentration of the two essential NMD factors Upf2 and Upf3b [17]. We therefore tested if such EJC-enhanced NMD of a miniμ mRNA can also be suppressed by folding back the poly(A) tail (Figures 4 and S5). To this end, we generated a FB construct with a PTC located in the fourth of six exons (miniμ ter310 FB, Figure 4A). After splicing, the mRNA of this minigene construct is expected to harbor two EJCs downstream of the PTC, and the corresponding NFB control mRNA (ter310 NFB) should therefore be subject to efficient EJC-enhanced NMD. Indeed, the mRNA half-life of control construct ter310 NFB was only 1.92 h (Figure 4B) and the steady-state mRNA level only 1.1% of the corresponding WT NFB mRNA (Figure 4C), indicative of efficient EJC-enhanced NMD. In contrast, the mRNA of the PTC-containing FB construct (ter310 FB) was reduced only to 30%–40% of WT NFB, and significantly stabilized, indicated by an average half-life of 4.69 h (Figure 4B). Importantly, the intramolecular base pairing by itself did not stabilize the mRNA when it did not position the poly(A) tail close to the PTC (ter310 SL), and mutations in the base pairing region of ter310 FB reverted the transcript back into an NMD substrate (Figure S4D–S4F). Rather than fitting a simple exponential decay curve, we noticed that ter310 NFB and ter310 SL mRNA exhibit a fast initial decay rate that slows down at lower mRNA levels. The reason for this apparently bi-phasic decay kinetics is currently not known. Because it is not observed with EJC-independent NMD reporters, it may reflect mechanistic differences between EJC-independent and EJC-enhanced NMD (see Discussion). Consistent with the result from the decay assay, NMD inhibition by RNAi-mediated Upf1 depletion (Figure 4C and 4D) or treatment of the cells with the translation inhibitor cycloheximide (Figure S5) elevated the mRNA levels of the NMD substrates ter310 NFB and ter310 SL by a factor of 10–20; ter310 FB behaved like WT NFB. We conclude that positioning of the poly(A) tail near a PTC efficiently suppresses both EJC-independent and EJC-enhanced NMD. The latter result shows that the NMD-inhibiting signal (poly(A) tail proximity) efficiently competes with the NMD-promoting signal (the downstream EJC), resulting in a strong attenuation of NMD.

### PABPC1 Is an NMD Antagonizing Signal

**Figure 5:**
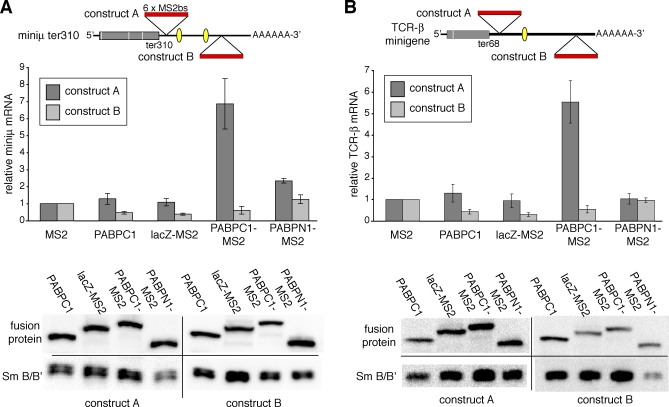
Tethering PABPC1 Nearby a PTC Suppresses NMD (A and B, upper panels) Shows schematic illustration of PTC-containing reporter genes miniμ (A) and TCRβ (B). A cassette comprising six MS2 binding sites (marked in red) was inserted about 50 nucleotides downstream of a PTC in construct A or further away in construct B. (A and B, middle panels) Relative mRNA levels of construct A (dark bars) and construct B (light bars), normalized to a cotransfected rGPx-1 gene, were measured by RT-qPCR. Average mRNA levels and SD were derived from two independent experiments with two qPCR runs each. (A and B, lower panels) The expression of the fusion proteins were analyzed by immunoblotting using monoclonal mouse α-HA antibody. Detection of SmB/B' served as loading control.

But which constituent of the poly(A) tail has the capacity to suppress NMD? It was recently shown for S. cerevisiae [18] and for *Drosophila* cell lines [19] that the poly(A)-binding protein inhibits NMD when tethered near the PTC on a NMD reporter transcript. To test if tethered poly(A)-binding protein inhibits NMD also in human cells, we tested both the nuclear and the cytoplasmic poly(A)-binding proteins (PABPN1 and PABPC1) expressed as N-terminal fusions to an HA-tagged variant of the MS2 coat protein and assessed their effect on miniμ and TCR-β NMD substrates that harbor six MS2 binding sites either about 50 nucleotides downstream of the PTC (constructs A) or further away as a control (constructs B, Figure 5). Western blotting confirmed comparable expression levels of the different fusion proteins. Tethering of PABPC1 caused a strong increase in both reporter mRNAs when tethered nearby the PTC, indicative for NMD suppression. In contrast, tethering of PABPN1, the MS2 domain alone, PABPC1 expression without the MS2 domain, or a fragment of β-galactosidase with similar mass as PABPC1 did not significantly stabilize the reporter mRNAs. Thus, as in D. melanogaster, only the cytoplasmic but not the nuclear PABP inhibited NMD when located in the vicinity of the PTC [19].

## Discussion

### Evidence for an Evolutionarily Conserved Mechanism for PTC Recognition

In summary, our results demonstrate that in human cells the proximity of PABPC1 provides an important signal for defining a translation termination event as “correct” and prevents degradation of the mRNA by NMD. Vice versa, translation termination too distant from PABPC1 lacks this signal, and as a consequence, NMD ensues. Our results are entirely consistent with models previously proposed for NMD in S. cerevisiae [18,21,28] and strongly argue that the basic mechanism for PTC recognition is much more conserved among eukaryotes than previously assumed. In essence, the current data suggest that the two antagonizing signals (PABPC1 proximity and Upf1–3 recruitment, respectively) determine if a translation termination event is defined as premature or correct. The recent characterization of PTC124, a small chemical entity that selectively induces ribosomal readthrough of premature but not normal TCs [29], further supports the idea of a mechanistic difference between translation termination at a PTC and termination at a “normal” TC.

Our finding that NMD suppression mediated through the poly(A) tail gradually declines with increasing distance between the TC and the poly(A) tail (Figure 3) is consistent with evidence suggesting that Upf1 and PABPC1 competes for interaction with release factor eRF3 bound to the ribosome at the TC [30]. Furthermore, our “distance model” provides a possible explanation for the reported distance effect of PABPC1 tethering to a β-globin NMD reporter transcript [31]. Consistent with our results on miniμ (Figures 3 and 5), NMD of this β-globin mRNA was suppressed more efficiently by tethering PABC1 45 nucleotides downstream of the PTC than tethering it 132 nucleotides downstream of the PTC [31]. The postulated requirement for correct translation termination to occur within a certain maximal physical distance from PABPC1 could also explain why PTCs near the start codon fail to trigger efficient NMD (Figure 1A and [32]), assuming that the start codon and the poly(A) tail are located in spatial proximity due to the interaction between eIF4G and PABPC1 [22].

### Mammalian EJCs Have Evolved to Function as NMD Enhancers

Our data further show that in mammals, the EJC is not required for PTC recognition, as in C. elegans and D. melanogaster [20,33]. But unlike C. elegans and D. melanogaster, where the EJC does not appear to affect NMD at all, the EJC plays an important role as an enhancer of NMD in mammals. The comparison between NMD of miniμ ter440 and the miniμ ter440 C3/C4 (3′-most intron deleted) represents an example for such an EJC-mediated enhancement of NMD [17]. The simplest mechanistic explanation for the NMD-enhancing effect of EJCs is that they accelerate SMG1/Upf2/Upf3-dependent phosphorylation of Upf1 by locally concentrating Upf2 and Upf3 [34]. We demonstrated here that even in a situation of such EJC-enhanced NMD, positioning the poly(A) tail between the PTC and the EJC still strongly suppressed NMD (Figure 4), indicating that the translation termination-promoting signal in this position still efficiently competed with NMD-promoting events. Confirming our result and showing that PABPC1 is necessary for this NMD suppression, Ivanov and colleagues found that artificially inducing NMD by tethering the EJC factor Y14 into the 3′ UTR of a β-globin reporter mRNA was suppressed by tethering PABPC1 between the TC and Y14 [35].

Noteworthy in the context of suppressing EJC-enhanced NMD, the mRNA decay kinetics of the reporter constructs in Figure 4B do not fit a simple exponential decay curve, but rather these transcripts seem to be degraded in an apparently bi-phasic mode. The initially fast decay rate might reflect the NMD enhancing effect of the EJC during the pioneer round of translation [25], whereas the second, somewhat slower decay rate would signify EJC-independent decay. We hypothesize that in mammals, where a large number of nonsense transcripts is produced by extensive alternative splicing [36], the EJC has evolved to function as an enhancer of NMD by locally concentrating Upf2 and Upf3b nearby terminating ribosomes and thereby tilting the balance of the two antagonizing signals toward NMD.

### Implications for Disease-Associated Mutations

Collectively, these data have potentially important clinical implications. Our findings predict that mutations leading to extended 3′ UTRs, such as poly(A) site mutations or sequence insertions into the 3′ UTR, constitute a so far overlooked group of NMD substrates that may explain the molecular mechanism of certain genetic diseases. For such mutations, treatments with readthrough-promoting drugs like PTC124 [29] would not be suitable, because PTC124 does not stabilize the mRNA and would lead to the synthesis of C-terminally extended wild-type protein. In contrast, mRNA stabilization by a FB strategy as described here would augment wild-type protein levels and therefore represent a putative gene-specific therapeutic approach, provided the FB can be induced in trans.

### Posttranscriptional Gene Regulation by Means of NMD

**Figure 6:**
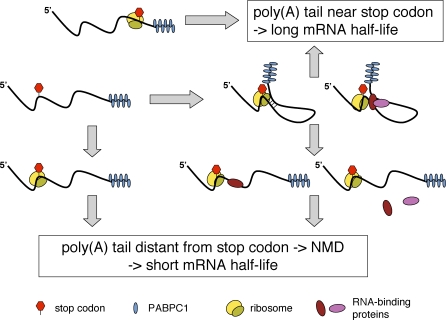
Model for Posttranscriptional Gene Regulation by Spatial Rearrangement of the 3′ UTR Proper translation termination requires a termination-promoting signal from the poly(A) tail. When the ribosome terminates close enough to the poly(A) tail to receive this signal no NMD occurs, and the mRNA remains intact. If the physical distance between the stop codon and the poly(A) tail is too large to allow transmission of the termination-promoting signal, NMD ensues, resulting in a short half-life and low steady-state level of the mRNA. The physical distance between the stop codon and the poly(A) tail depends on the 3-D structure of the 3′ UTR. The 3′ UTR structure can be reconfigured by altering (i) intramolecular base pairing, (ii) interaction of the mRNA with RNA-binding proteins, and (iii) interactions among the involved proteins through posttranslational modifications.

It has not escaped our notice that the new, unified NMD model we postulate immediately suggests a possible mechanism for posttranscriptional regulation of a wide variety of genes by NMD. It is well documented that 3′ UTRs, many of which comprise several thousand nucleotides in mammals, serve as binding sites for numerous factors that regulate mRNA translation or stability [26,27]. We postulate that by binding to their target transcript, many of these factors alter the tertiary structure of the 3′ UTR, thereby changing the local environment for translation termination (i.e., the physical distance between the TC and the poly(A) tail), which in turn will amend the transcript's half-life (Figure 6). 3′ UTR-binding factors can change the 3-D 3′ UTR configuration by masking mRNA sequences otherwise engaged in intramolecular base pairing, or by interacting with each other and thereby looping out mRNA sequence in-between. Protein-protein and protein-RNA interactions can be regulated through signal transduction pathways by posttranslational modification of the involved RNA-binding proteins. Furthermore, this NMD-dependent posttranscriptional gene regulation can also be modulated through transcript-specific RNA-binding proteins with intrinsic NMD-promoting or translation termination–promoting activity that binds into the proximity of the TC. For example, the RNA-binding protein Staufen has been reported to bind the 3′ UTR of a few specific mRNAs and to induce their rapid degradation by directly recruiting Upf1 [37,38]. Although it remains to be further investigated to which extent cells use this gene regulation pathway, the surprisingly large number of physiological transcripts detected by microarray analysis that raise in levels upon Upf1 knockdown indicates that it might be widespread [39–43]. A central prediction of this NMD-mediated gene regulation mechanism is that it depends on ongoing translation and that one would expect the set of transcripts affected by Upf1 depletion to vary in a tissue-specific manner, during development and differentiation, and by environmental cues in general. This might explain why the sets of transcripts affected by Upf1 depletion in the different microarray studies showed only limited overlap [39–43]. To test the postulated mode of gene regulation by spatial remodeling of the 3′ UTR more directly, development of techniques that allow in vivo measurements of the physical distance between two molecules and improved predictions of mRNA folding in the presence of RNA-binding proteins will be necessary.

## Materials and Methods

### Plasmids.

The Ig-μ minigenes miniμWT, ter32, ter57, ter73, ter108, ter310, ter440, ter452, and ter459 are described elsewhere [44]. Nonsense mutations at amino acid positions 190 (CAG to TAG), 472 (TAC to TAG), and 517 (TAT to TAA) were generated by PCR-mediated site-directed mutagenesis using QuikChange XL Site-Directed Mutagenesis Kit (Stratagene). The sequence of the entire ORF in each construct was confirmed by sequencing. For the miniμ constructs with extended 3′ UTRs, a unique Cla1 site 90 bp downstream of the TC and a unique Spe1 site 16 bp upstream of the poly(A) signal were generated in pβminiμ WT C3/C4 [17] by site-directed mutagenesis. The Ig-μ minigenes with extended 3′ UTR were generated by inserting 600-bp long PCR fragments of the β-lactamase (Amp) gene into the Spe1 site (+600) or additionally, a second, similar PCR fragment into the Cla1 site (+1,200). For the foldback constructs, a unique Spe1 site was generated in pβminiμWT 16 bp upstream of the poly(A) signal by site-directed mutagenesis and its BamH1 fragment was exchanged with the BamH1 fragment of miniμ ter440 C4/H4 or miniμWT C4/H4 [17]. Insertion into the Spe1 site of these plasmids of a ds-oligo with the sequence 5′-ctagCATTGGGTTTTGAGATGAATTTCTTC-3′ gave miniμ ter440 C3/H4 FB and miniμ WT C3/H4 FB, and insertion of the ds-oligo in the opposite orientation gave miniμ ter440 C3/H4 NFB and miniμ WT C3/H4 NFB, respectively. The plasmids miniμ ter440 FB2, FB3, FB4, and FB5 were generated correspondingly by inserting the sequence 5′-ctagTGCCAGACATAGCTAATGTAATCTGAAAC-3′ (FB2), 5′-ctagCCTGGATGTTGTCACGCAAGAC-3′ (FB3), 5′-ctagAGGAACACCTTCAGCACACCAC-3′ (FB4), and 5′-ctagCAAAACCAGTGACGTTTGAATGG-3′ (FB5) into the Spe1 site. The plasmids miniμ ter310 FB, ter310 NFB, and WT NFB were generated analogously by inserting the sequence 5′-ctagTCGATTTCAGAGATGGTAAGTGTGC-3′ or the reverse complement into Spe1 of miniμ ter310 and miniμ WT. The plasmid pβminiμter310 SL was generated by insertion of the sequence above into the BsrG1 site of exon C2. The nucleotide substitutions to create the mutant FB constructs, numbered relative to the AUG start codon, were C964A, T965G, C968G, C969G, C974A, T975G, and G976C for miniμ ter310 FB and T1369G, T1370G, C1371A, T1375G, C1376A, C1381G, and C1382A for miniμ C3/H4 ter440 FB (sequence available upon request). pBS β-globin WT was previously described [45]. A unique Mlu1 site two nucleotides downstream of the TC was generated by site-directed mutagenesis, and a 577-bp PCR fragment of the β-lactamase (Amp) gene with flanking Mlu1 sites was inserted to generate pBS β-globin WT +577. For the half-life measurements, the entire reporter constructs from Kpn1 (in the 5′ UTR) to about 190 bp downstream of the polyA signal were inserted into Kpn1–Xho1 of pTRE-Tight (Clontech).

For the miniμ and TCRβ reporter gene constructs used in the tethering assay, a fragment encoding six MS2 binding sites from pcβwt β2–3MS2 [11] was PCR-amplified with flanking BsrG1 sites and inserted either into the BsrG1 site of exon C2 (giving the construct A) or into the BsrG1 site of exon C4 (giving the construct B) of miniμ ter310. For TCR-β the six MS2 binding sites were introduced in the Stu1 (construct A) or BamH1 (construct B) site of the TCR-β construct pβ434 [46]. The control plasmid containing the MS2 binding domain and a HA-tag was derived from pCMV-H2b-MS2-HA by replacing the H2b encoding Kpn1–BamH1 fragment with the annealed oligos 5′-CACCATG-3′ and 5′-GATCCATGGTGGTAC-3′ to introduce the Kosak consensus sequence. PABPC1 and PABPN1 were PCR-amplified from pETNHis-hPABPC1 (obtained from Elmar Wahle, University of Halle-Wittenberg, Germany) and pcDNA3-hPABPN1-HA [47] with flanking Kpn1–BamH1 sites and cloned into Kpn1–BamH1 of pCMV-H2b-MS2-HA to give pCMV-PABPC1-MS2-HA and pCMV-PABPN1-MS2-HA, respectively. The MS2-HA fragment was removed by digestion with BamH1 and Not1 and the HA-tag was then reintroduced by ds-oligos to generate pCMV-PABPC1-HA. The C-terminal part of the lacZ gene (1,910 bp) was PCR-amplified and inserted into Kpn1–BamH1 as described above. pSUPERpuro-hUpf1 and pSUPERpuro-scrambled constructs were described elsewhere [17,48]. pSUPERpuro-hUpf2 was obtained by transfering the EcoR1–HindIII fragment of pSUPER-hUpf2 [43] into pSUPERpuro. pSUPERpuro-hUpf3b-1 and pSUPERpuro-hUpf3b-2 were generated by insertion of double-stranded oligos encoding for short hairpin RNAs (shRNAs) into pSUPERpuro between the BglII and HindIII sites as described previously [49]. The two target sequences of hUpf3b were 5′-GGTGGTAATTCGAAGATTA-3′ (pSUPERpuro-hUpf3b-1) and 5′-CGAGATCAGGAGCGCATAC-3′ (pSUPERpuro-hUpf3b-2).

### Cell culture and cycloheximide treatment.

HeLa cells were grown in Dulbecco's modified Eagle's medium (DMEM, Invitrogen), supplemented with 10% heat-inactivated fetal calf serum (FCS), 100 U/mL penicillin, and 100 μg/mL streptomycin (Amimed). For the experiment in Figure S5, 44 h after transfection of the reporter plasmids, 100 μg/mL cycloheximide was added for 4 h before RNA isolation.

### Half-life measurements.

The Tet-Off Advanced Transactivator (tTA-Advanced) was stably integrated into the genome of HeLa cells according to the manufacturer's protocol (Tet-Off Advanced Inducible Gene Expression System, Clontech) and a cell clone with high tTA-Advanced expression was selected for further experiments. To determine decay kinetics of the miniμ reporter mRNAs, 2 × 10^5 ^ per well of the tTA-Advanced expressing cells were seeded in 6-well plates. The next day, two wells for each time course were transfected with 100 ng pTRE-Tight miniμ reporter plasmid and 100 ng pBS β-globin WT plasmid (for normalization) per well, using 2 μl DreamFect (OZ Biosciences) according to the manufacturer's protocol. On the following day, the cells of these two wells were split into six wells. Time course was started 40 h after transfection by adding 1 μg/ml doxycycline (Sigma) to each well and harvesting the cells after 0, 1, 2, 4, 6, and 8 h.

### Transient transfections and quantitative real-time RT-PCR.

2–3 × 10^5^ HeLa cells were seeded in 6-well plates and transfected the next day with 100–150 ng reporter plasmid, 100–150 ng plasmid encoding a gene for normalization, and 2–4 μL DreamFect (OZ Biosciences) according to manufacturer's protocol. For normalization, pBS β-globin WT was used in all experiments except for Figures 5 and S2, where pCMVrGPx1-TGC [50] was used instead. For the tethering experiments in Figure 5, 300 ng MS2 fusion protein-encoding plasmid was cotransfected with 100 ng of reporter plasmid, and the cells were harvested 52 h after transfection. Total cellular RNA was isolated using “Absolutely RNA RT-PCR Miniprep Kit” (Stratagene) and 1 μg RNA was reverse transcribed in 50 μL Stratascript first strand buffer in the presence of 0.4 mM dNTPs, 300 ng random hexamers, 40 U RNasIn (Promega), and 50 U Stratascript reverse transcriptase (Stratagene) in Figures 1A, 2C, 4C, and S2. For Figures 1B–1D, 2B, 3, 4B, 5, S1, S4, and S5, 1 μg RNA was reverse transcribed in 20 μL StrataScript 6.0 RT buffer in the presence of 1 mM dNTPs, 300 ng random hexamers, 40 U RNasIn (Promega), and 50 U StrataScript 6.0 reverse transcriptase (Stratagene) according to manufacturer's protocol. Real-time RT-PCR was performed as previously described [17].

### RNAi.

Knockdown of hUpf1, hUpf2, and hUpf3b was induced by transfection of pSUPERpuro plasmids targeting two different sequences in hUpf1 [48], one sequence in hUpf2 [43], or two different sequences in hUpf3b (see above), respectively. Starting 24 h after transfection, untransfected cells were eliminated by culturing the cells in the presence of 1.5 μg/mL puromycin for 48 h. Cells were then washed in PBS and incubated in puromycin-free medium for another 24 h. Total cellular RNA was isolated and whole cell lysates for Western blotting were prepared 96 h post transfection. The efficiency of the knockdown was assessed on the mRNA level by real-time RT-PCR (unpublished data) and on the protein level by Western blotting.

### Northern blot analysis.

Total cellular RNA (10 μg) was separated on a 1.2% agarose gel containing 1× MOPS and 1% formaldehyde. RNA was transferred to positively charged nylon membrane (Roche) in 0.5× MOPS by 1-h wet blotting in a genie blotter (Idea Scientific). Following UV crosslinking of the RNA to the nylon filter, pre-hybridization and hybridization of the blot in Figure 1B was carried out in 6× SSC, 5× Denhardt's reagent, and 0.5% SDS with 50 μg/mL denatured salmon sperm DNA and 100 μg/mL denatured calf thymus DNA at 60 °C. For hybridization, 100 ng μter310 and 20 ng β-globin DNA was labeled with α-^32^P-dCTP using the Ready-To-Go DNA-Labeling Kit (Amersham). The blot in Figure S3 was hybridized with an in vitro–transcribed, α-^32^P-UTP-labeled antisense miniμ RNA probe in ULTRAHyb buffer (Ambion) at 68 °C. After overnight hybridization, membranes were washed twice with 2× SSC/0.2% SDS and twice with 0.2× SSC/0.1% SDS at 60 °C before exposure to a PhosphorImager screen.

### Immunoblotting.

Whole cell lysates corresponding to 0.37 × 10^4^ – 2 × 10^5^ cells per lane were electrophoresed on a 10% SDS-PAGE. Proteins were transferred to Optitran BA-S 85 reinforced nitrocellulose (Schleicher and Schuell) and probed with 1:2,500 diluted polyclonal rabbit anti-hUpf1, anti-hUpf2, or anti-hUpf3b antiserum [11], 1:1,000 diluted monoclonal mouse anti-lamin A/C (Santa Cruz Biotechnology) or anti-HA antibody (Roche), 1:400 diluted supernatant of the mouse hybridoma cell line Y12, which produces a monoclonal antibody against the human Sm B/B'proteins [51]. 1:2,500 diluted HRP-conjugated anti-rabbit IgG or HRP-conjugated anti-mouse IgG (Promega) was used as secondary antibody. ECL+ Plus Western blotting detection system (Amersham) was used for detection and signals were visualized on a Luminescent Image Analyzer LAS-1000 (Fujifilm).

## Supporting Information

Figure S1 Monitoring of the Knockdown Efficacy of Upf1, Upf2, and Upf3b at the mRNA Level From the RNA samples of Figure 1D, relative mRNA levels of Upf1, Upf2, and Upf3b, normalized to endogenous GAPDH mRNA, were measured by RT-qPCR using the TaqMan assay Hs00161289_m1, Hs00210187_m1, Hs00224875_m1, and 432-6317E from Applied Biosystems. Average values of two qPCR runs are shown.
(214 KB PDF)

Figure S2 Extension of the 3′ UTR Converts the β-globin WT mRNA into a NMD Substrate Relative β-globin mRNA levels from constructs with (+577) or without (control) a 3′ UTR extension, normalized to the mRNA levels of a cotransfected rGPx-1 gene, were determined in cells depleted (Upf1 k.d.) or not (scr k.d.) for Upf1. The effect of Upf1 knockdown on β-globin mRNA is shown in the histogram, and the efficacy of Upf1 depletion was monitored by Western blotting (lower panel). Average values and SD are from two independent experiments with three qPCR runs each. k.d., knockdown.
(362 KB PDF)

Figure S3 Detection of Miniμ mRNA by Northern Blot Analysis (A) Miniμ C3H4 mRNA of FB and control NFB constructs from the RNA samples with Upf1 knockdown analyzed in Figure 2C.
(B) Miniμ mRNA of the constructs shown in Figure 4A. RNA samples of cycloheximide-treated cells (analyzed in Figure S5) were used.
(336 KB PDF)

Figure S4 Mutant FB Constructs Do Not Suppress NMD Mutations in the sequence downstream of the PTC were introduced to abolish the folding back of the poly(A) tail of the miniμ C3/H4 ter440 FB construct (A) and of the miniμ ter310 FB construct (D). The mutations are depicted in red. (B) and (E) Relative miniμ mRNA levels normalized to β-globin WT mRNA from a cotransfected plasmid were measured by RT-qPCR in cells depleted for Upf1 (Upf1 k.d.) or not (scr k.d.). Average mRNA levels and SD from one experiment with three RT-qPCR runs are shown and displayed as in the corresponding Figures 2C and 4C. (C) and (F) The efficacy of the Upf1 knockdown was assessed by Western blotting. SmB/B' was detected as loading control.
(622 KB PDF)

Figure S5 Suppression of EJC-Enhanced NMD by Poly(A) Tail FB Relative miniμ mRNA levels from the constructs indicated in Figure 4A, normalized and displayed as in Figure 4C, from cells treated (+CHX) or not (control) with 100 μg/mL cycloheximide for 4 h before RNA isolation. Average values and SD of five qPCR measurements from two independent experiments are shown.
(241 KB PDF)

## Abbreviations

EJC: exon junction complex
FB: foldback
NFB: no foldback
NMD: nonsense-mediated mRNA decay
PABP: poly(A)-binding protein
PTC: premature termination codon
RT-PCR: reverse-transcriptase PCR
RT-qPCR: reverse-transcriptase quantitative PCR
TC: termination codon
UTR: untranslated region

## References

[pbio-0060092-b001] Isken O, Maquat LE (2007). Quality control of eukaryotic mRNA: safeguarding cells from abnormal mRNA function. Genes Dev.

[pbio-0060092-b002] Chang YF, Imam JS, Wilkinson MF (2007). The nonsense-mediated decay RNA surveillance pathway. Annu Rev Biochem.

[pbio-0060092-b003] Frischmeyer PA, Dietz HC (1999). Nonsense-mediated mRNA decay in health and disease. Mol Genet Hum.

[pbio-0060092-b004] Holbrook JA, Neu-Yilik G, Hentze MW, Kulozik AE (2004). Nonsense-mediated decay approaches the clinic. Nat Genet.

[pbio-0060092-b005] Rehwinkel J, Raes J, Izaurralde E (2006). Nonsense-mediated mRNA decay: target genes and functional diversification of effectors. Trends Biochem Sci.

[pbio-0060092-b006] Culbertson MR, Leeds PF (2003). Looking at mRNA decay pathways through the window of molecular evolution. Curr Opin Genet Dev.

[pbio-0060092-b007] Bhattacharya A, Czaplinski K, Trifillis P, He F, Jacobson A (2000). Characterization of the biochemical properties of the human Upf1 gene product that is involved in nonsense-mediated mRNA decay. RNA.

[pbio-0060092-b008] Weng Y, Czaplinski K, Peltz SW (1996). Genetic and biochemical characterization of mutations in the ATPase and helicase regions of the Upf1 protein. Mol Cell Biol.

[pbio-0060092-b009] Cheng Z, Muhlrad D, Lim MK, Parker R, Song H (2007). Structural and functional insights into the human Upf1 helicase core. EMBO J.

[pbio-0060092-b010] He F, Brown AH, Jacobson A (1997). Upf1p, Nmd2p, and Upf3p are interacting components of the yeast nonsense-mediated mRNA decay pathway. Mol Cell Biol.

[pbio-0060092-b011] Lykke-Andersen J, Shu MD, Steitz JA (2000). Human Upf proteins target an mRNA for nonsense-mediated decay when bound downstream of a termination codon. Cell.

[pbio-0060092-b012] Mendell JT, Medghalchi SM, Lake RG, Noensie EN, Dietz HC (2000). Novel Upf2p orthologues suggest a functional link between translation initiation and nonsense surveillance complexes. Mol Cell Biol.

[pbio-0060092-b013] Serin G, Gersappe A, Black JD, Aronoff R, Maquat LE (2001). Identification and characterization of human orthologues to Saccharomyces cerevisiae Upf2 protein and Upf3 protein (Caenorhabditis elegans SMG-4). Mol Cell Biol.

[pbio-0060092-b014] Yamashita A, Kashima I, Ohno S (2005). The role of SMG-1 in nonsense-mediated mRNA decay. Biochim Biophys Acta.

[pbio-0060092-b015] Lejeune F, Maquat LE (2005). Mechanistic links between nonsense-mediated mRNA decay and pre-mRNA splicing in mammalian cells. Curr Opin Cell Biol.

[pbio-0060092-b016] Behm-Ansmant I, Izaurralde E (2006). Quality control of gene expression: a stepwise assembly pathway for the surveillance complex that triggers nonsense-mediated mRNA decay. Genes Dev.

[pbio-0060092-b017] Buhler M, Steiner S, Mohn F, Paillusson A, Muhlemann O (2006). EJC-independent degradation of nonsense immunoglobulin-mu mRNA depends on 3′ UTR length. Nat Struct Mol Biol.

[pbio-0060092-b018] Amrani N, Ganesan R, Kervestin S, Mangus DA, Ghosh S (2004). A faux 3′-UTR promotes aberrant termination and triggers nonsense-mediated mRNA decay. Nature.

[pbio-0060092-b019] Behm-Ansmant I, Gatfield D, Rehwinkel J, Hilgers V, Izaurralde EA (2007). Conserved role for cytoplasmic poly(A)-binding protein 1 (PABPC1) in nonsense-mediated mRNA decay. EMBO J.

[pbio-0060092-b020] Longman D, Plasterk RH, Johnstone IL, Caceres JF (2007). Mechanistic insights and identification of two novel factors in the C. elegans NMD pathway. Genes Dev.

[pbio-0060092-b021] Amrani N, Sachs MS, Jacobson A (2006). Early nonsense: mRNA decay solves a translational problem. Nat Rev Mol Cell Biol.

[pbio-0060092-b022] Wells SE, Hillner PE, Vale RD, Sachs AB (1998). Circularization of mRNA by eukaryotic translation initiation factors. Mol Cell.

[pbio-0060092-b023] Lejeune F, Ranganathan AC, Maquat LE (2004). eIF4G is required for the pioneer round of translation in mammalian cells. Nat Struct Mol Biol.

[pbio-0060092-b024] Fortes P, Inada T, Preiss T, Hentze MW, Mattaj IW (2000). The yeast nuclear cap binding complex can interact with translation factor eIF4G and mediate translation initiation. Mol Cell.

[pbio-0060092-b025] Ishigaki Y, Li X, Serin G, Maquat LE (2001). Evidence for a pioneer round of mRNA translation. mRNAs subject to nonsense-mediated decay in mammalian cells are bound by CBP80 and CBP20. Cell.

[pbio-0060092-b026] Wickens M, Anderson P, Jackson RJ (1997). Life and death in the cytoplasm: messages from the 3′ end. Curr Opin Genet Dev.

[pbio-0060092-b027] Moore MJ (2005). From birth to death: the complex lives of eukaryotic mRNAs. Science.

[pbio-0060092-b028] Hilleren P, Parker R (1999). mRNA surveillance in eukaryotes: kinetic proofreading of proper translation termination as assessed by mRNP domain organization. RNA.

[pbio-0060092-b029] Welch EM, Barton ER, Zhuo J, Tomizawa Y, Friesen WJ (2007). PTC124 targets genetic disorders caused by nonsense mutations. Nature.

[pbio-0060092-b030] Singh G, Rebbapragada I, Lykke-Andersen JA (2008). Competition between stimulators and antagonists of Upf complex recruitment governs human nonsense-mediated mRNA decay. PLoS Biol.

[pbio-0060092-b031] Silva AL, Ribeiro P, Inacio A, Liebhaber SA, Romao L (2008). Proximity of the poly(A)-binding protein to a premature termination codon inhibits mammalian nonsense-mediated mRNA decay. RNA.

[pbio-0060092-b032] Silva AL, Pereira FJ, Morgado A, Kong J, Martins R (2006). The canonical UPF1-dependent nonsense-mediated mRNA decay is inhibited in transcripts carrying a short open reading frame independent of sequence context. RNA.

[pbio-0060092-b033] Gatfield D, Unterholzner L, Ciccarelli FD, Bork P, Izaurralde E (2003). Nonsense-mediated mRNA decay in Drosophila: at the intersection of the yeast and mammalian pathways. EMBO J.

[pbio-0060092-b034] Kashima I, Yamashita A, Izumi N, Kataoka N, Morishita R (2006). Binding of a novel SMG-1-Upf1-eRF1-eRF3 complex (SURF) to the exon junction complex triggers Upf1 phosphorylation and nonsense-mediated mRNA decay. Genes Dev.

[pbio-0060092-b035] Ivanov PV, Gehring NH, Kunz JB, Hentze MW, Kulozik AE (2008). Interactions between UPF1, eRFs, PABP, and the exon junction complex suggest an integrated model for mammalian NMD pathways. EMBO J.

[pbio-0060092-b036] Lewis BP, Green RE, Brenner SE (2003). Evidence for the widespread coupling of alternative splicing and nonsense-mediated mRNA decay in humans. Proc Natl Acad Sci U S A.

[pbio-0060092-b037] Kim YK, Furic L, Desgroseillers L, Maquat LE (2005). Mammalian Staufen1 recruits Upf1 to specific mRNA 3′ UTRs so as to elicit mRNA decay. Cell.

[pbio-0060092-b038] Kim YK, Furic L, Parisien M, Major F, DesGroseillers L (2007). Staufen1 regulates diverse classes of mammalian transcripts. EMBO J.

[pbio-0060092-b039] He F, Li X, Spatrick P, Casillo R, Dong S (2003). Genome-wide analysis of mRNAs regulated by the nonsense-mediated and 5′ to 3′ mRNA decay pathways in yeast. Mol Cell.

[pbio-0060092-b040] Lelivelt MJ, Culbertson MR (1999). Yeast Upf proteins required for RNA surveillance affect global expression of the yeast transcriptome. Mol Cell Biol.

[pbio-0060092-b041] Mendell JT, Sharifi NA, Meyers JL, Martinez-Murillo F, Dietz HC (2004). Nonsense surveillance regulates expression of diverse classes of mammalian transcripts and mutes genomic noise. Nat Genet.

[pbio-0060092-b042] Rehwinkel J, Letunic I, Raes J, Bork P, Izaurralde E (2005). Nonsense-mediated mRNA decay factors act in concert to regulate common mRNA targets. RNA.

[pbio-0060092-b043] Wittmann J, Hol EM, Jack HM (2006). hUPF2 silencing identifies physiologic substrates of mammalian nonsense-mediated mRNA decay. Mol Cell Biol.

[pbio-0060092-b044] Buhler M, Paillusson A, Muhlemann O (2004). Efficient downregulation of immunoglobulin mu mRNA with premature translation-termination codons requires the 5′-half of the VDJ exon. Nucleic Acids Res.

[pbio-0060092-b045] Thermann R, Neu-Yilik G, Deters A, Frede U, Wehr K (1998). Binary specification of nonsense codons by splicing and cytoplasmic translation. EMBO J.

[pbio-0060092-b046] Mohn F, Buhler M, Muhlemann O (2005). Nonsense-associated alternative splicing of T cell receptor beta genes: no evidence for frame dependence. RNA.

[pbio-0060092-b047] Tavanez JP, Calado P, Braga J, Lafarga M, Carmo-Fonseca M (2005). In vivo aggregation properties of the nuclear poly(A)-binding protein PABPN1. RNA.

[pbio-0060092-b048] Paillusson A, Hirschi N, Vallan C, Azzalin CM, Muhlemann O (2005). A GFP-based reporter system to monitor nonsense-mediated mRNA decay. Nucleic Acids Res.

[pbio-0060092-b049] Brummelkamp TR, Bernards R, Agami RA (2002). System for stable expression of short interfering RNAs in mammalian cells. Science.

[pbio-0060092-b050] Moriarty PM, Reddy CC, Maquat LE (1997). The presence of an intron within the rat gene for selenium-dependent glutathione peroxidase 1 is not required to protect nuclear RNA from UGA-mediated decay. RNA.

[pbio-0060092-b051] Lerner EA, Lerner MR, Janeway CA, Steitz JA (1981). Monoclonal antibodies to nucleic acid-containing cellular constituents: probes for molecular biology and autoimmune disease. Proc Natl Acad Sci U S A.

