# Supplementary figures and images for: Posttranscriptional Gene Regulation by Spatial Rearrangement of the 3′ Untranslated Region
Source: PLoS Biol. 2008 Apr 29;6(4):e92. doi: 10.1371/journal.pbio.0060092 (PMC2689704; doi:10.1371/journal.pbio.0060092)

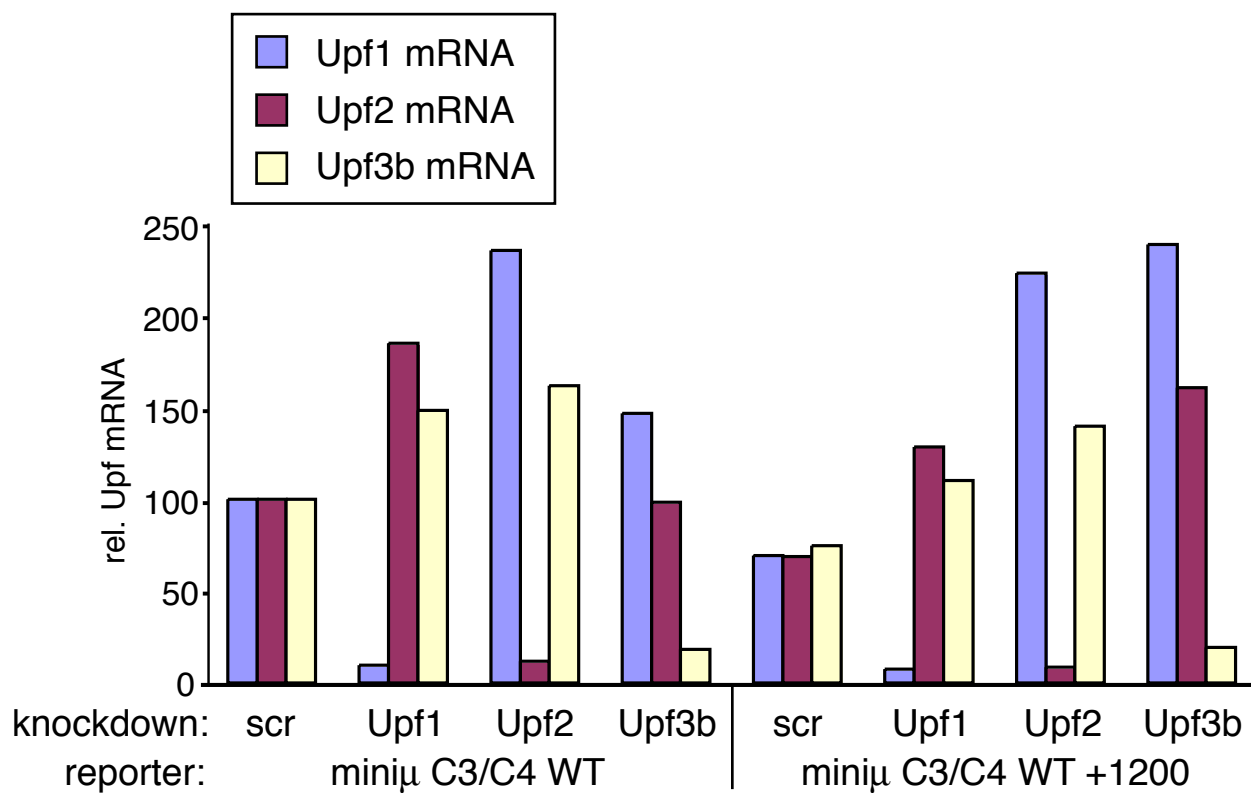

Supplement: Figure S1 — From the RNA samples of Figure 1D, relative mRNA levels of Upf1, Upf2, and Upf3b, normalized to endogenous GAPDH mRNA, were measured by RT-qPCR using the TaqMan assay Hs00161289_m1, Hs00210187_m1, Hs00224875_m1, and 432-6317E from Applied Biosystems. Average values of two qPCR runs are shown. (214 KB PDF) [file pbio.0060092.sg001.pdf]

Figure S2

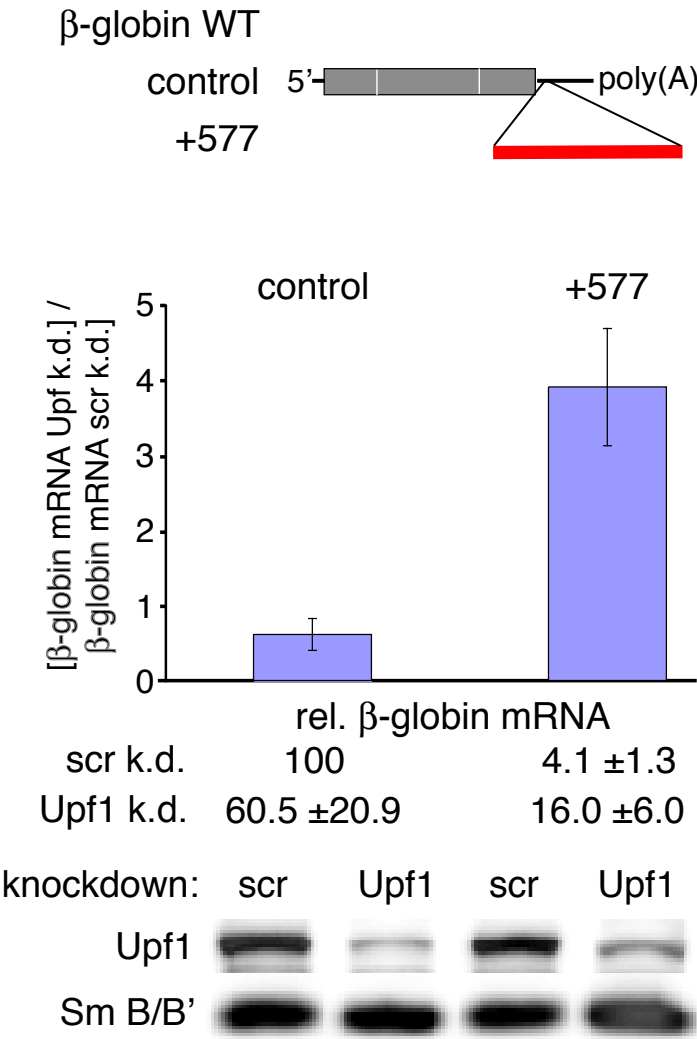

Supplement: Figure S2 — Relative β-globin mRNA levels from constructs with (+577) or without (control) a 3′ UTR extension, normalized to the mRNA levels of a cotransfected rGPx-1 gene, were determined in cells depleted (Upf1 k.d.) or not (scr k.d.) for Upf1. The effect of Upf1 knockdown on β-globin mRNA is shown in the histogram, and the efficacy of Upf1 depletion was monitored by Western blotting (lower panel). Average values and SD are from two independent experiments with three qPCR runs each. k.d., knockdown. (362 KB PDF) [file pbio.0060092.sg002.pdf]

Figure S3

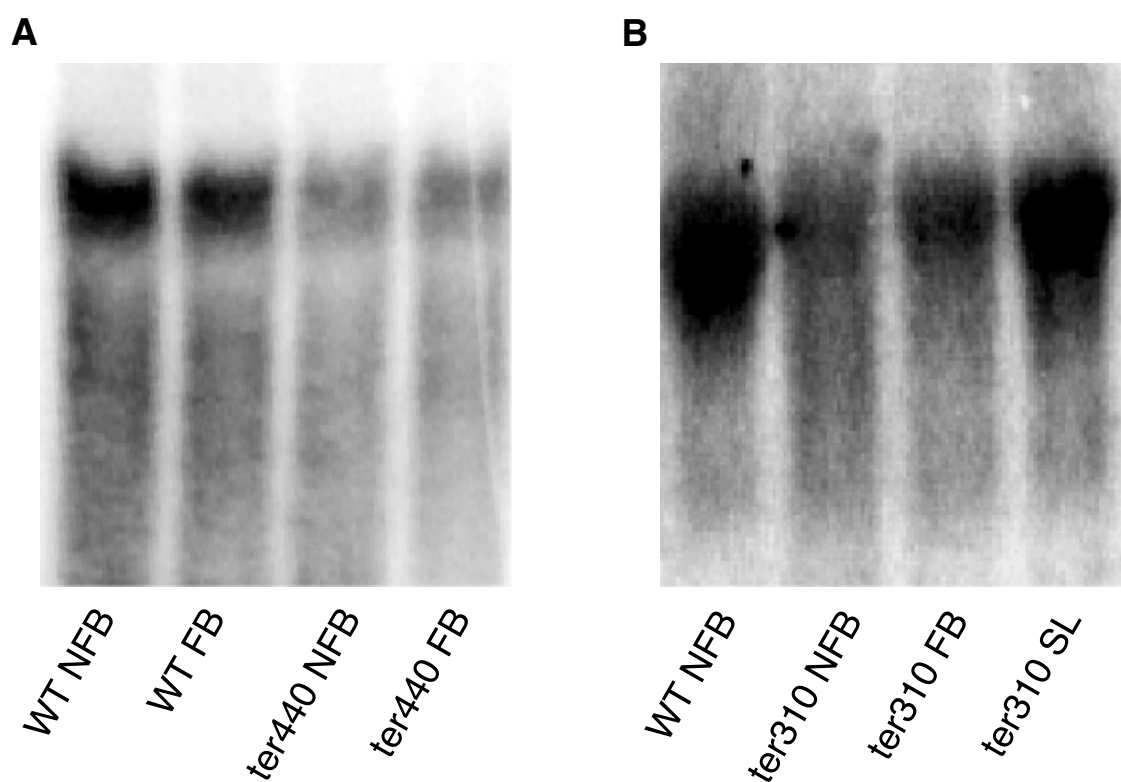

Supplement: Figure S3 — (A) Miniμ C3H4 mRNA of FB and control NFB constructs from the RNA samples with Upf1 knockdown analyzed in Figure 2C. (B) Miniμ mRNA of the constructs shown in Figure 4A. RNA samples of cycloheximide-treated cells (analyzed in Figure S5) were used. (336 KB PDF) [file pbio.0060092.sg003.pdf]

Figure S4

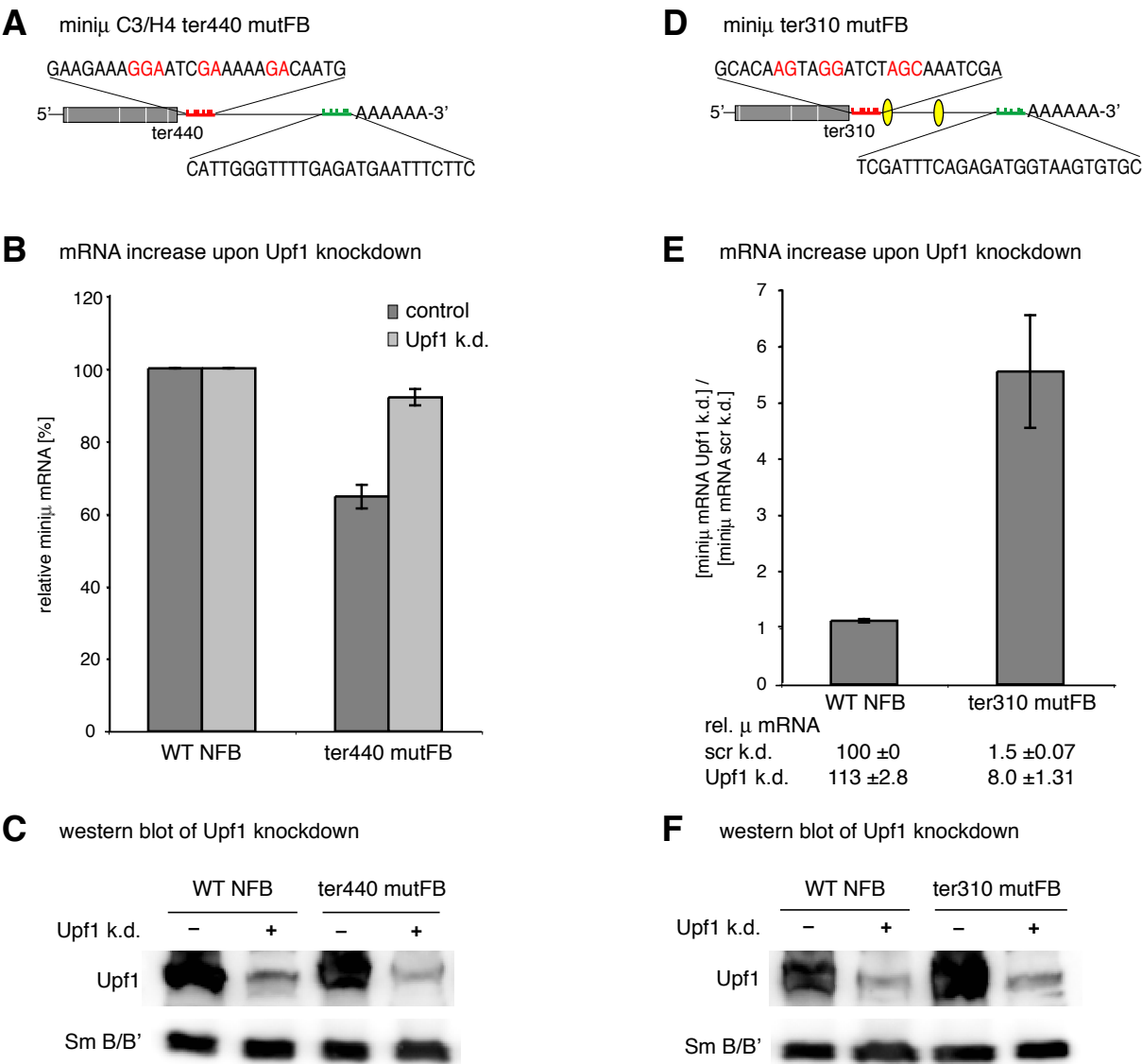

Supplement: Figure S4 — Mutations in the sequence downstream of the PTC were introduced to abolish the folding back of the poly(A) tail of the miniμ C3/H4 ter440 FB construct (A) and of the miniμ ter310 FB construct (D). The mutations are depicted in red. (B) and (E) Relative miniμ mRNA levels normalized to β-globin WT mRNA from a cotransfected plasmid were measured by RT-qPCR in cells depleted for Upf1 (Upf1 k.d.) or not (scr k.d.). Average mRNA levels and SD from one experiment with three RT-qPCR runs are shown and displayed as in the corresponding Figures 2C and 4C. (C) and (F) The efficacy of the Upf1 knockdown was assessed by Western blotting. SmB/B' was detected as loading control. (622 KB PDF) [file pbio.0060092.sg004.pdf]
