# Supplementary material for: Posttranscriptional Gene Regulation by Spatial Rearrangement of the 3′ Untranslated Region
Source: PLoS Biol. 2008 Apr 29;6(4):e92. doi: 10.1371/journal.pbio.0060092 (PMC2689704; doi:10.1371/journal.pbio.0060092)
Supplement: Figure S5 — Relative miniμ mRNA levels from the constructs indicated in Figure 4A, normalized and displayed as in Figure 4C, from cells treated (+CHX) or not (control) with 100 μg/mL cycloheximide for 4 h before RNA isolation. Average values and SD of five qPCR measurements from two independent experiments are shown. (241 KB PDF) [file pbio.0060092.sg005.pdf]

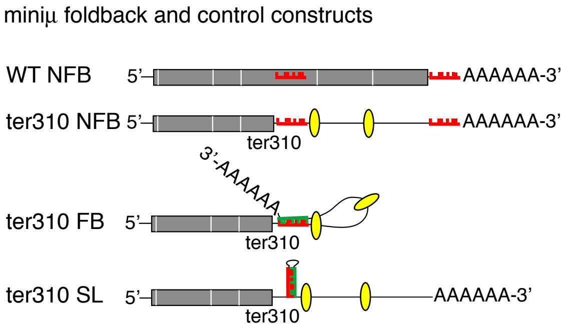

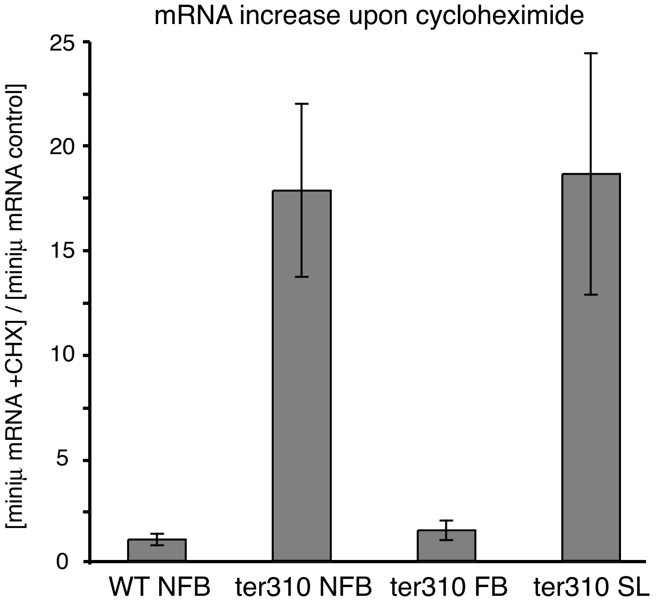

rel. μ mRNA				
control	100 ±0	1.8 ±0.3	42.0 ±9.4	1.4 ±0.26
+ CHX	102.8 ±27.1	32.8 ±4.6	62.2 ±14.7	26.8 ±6.8
